# Probiotics Supplementation Improves Intestinal Permeability, Obesity Index and Metabolic Biomarkers in Elderly Thai Subjects: A Randomized Controlled Trial

**DOI:** 10.3390/foods11030268

**Published:** 2022-01-19

**Authors:** Chaiyavat Chaiyasut, Bhagavathi Sundaram Sivamaruthi, Narissara Lailerd, Sasithorn Sirilun, Suchanat Khongtan, Pranom Fukngoen, Sartjin Peerajan, Manee Saelee, Khontaros Chaiyasut, Periyanaina Kesika, Phakkharawat Sittiprapaporn

**Affiliations:** 1 Innovation Center for Holistic Health, Nutraceuticals, and Cosmeceuticals, Faculty of Pharmacy, Chiang Mai University, Chiang Mai 50200, Thailand; chaiyavat@gmail.com (C.C.); sivamaruthi.b@cmu.ac.th (B.S.S.); suchanat_k@cmu.ac.th (S.K.); pranom_fukngoen@cmu.ac.th (P.F.); manee_saelee@cmu.ac.th (M.S.); kesika.p@cmu.ac.th (P.K.); 2 Office of Research Administration, Chiang Mai University, Chiang Mai 50200, Thailand; 3 Department of Physiology, Faculty of Medicine, Chiang Mai University, Chiang Mai 50200, Thailand; narissara.lailerd@cmu.ac.th; 4 Department of Pharmaceutical Sciences, Faculty of Pharmacy, Chiang Mai University, Chiang Mai 50200, Thailand; 5 Health Innovation Institute, Chiang Mai 50200, Thailand; s.peerajan@gmail.com; 6 Institute of Research and Development, Chiang Mai Rajabhat University, Chiang Mai 50300, Thailand; khontaros_cha@cmru.ac.th; 7 Neuropsychological Research Laboratory, Department of Anti-Aging and Regenerative Science, School of Anti-Aging and Regenerative Medicine, Mae Fah Luang University, Bangkok 11120, Thailand

**Keywords:** probiotics, *Lactobacillus*, *Bifidobacterium*, intestinal permeability, cholesterol

## Abstract

Intestinal integrity prevents the diffusion of allergens, toxins, and pathogens from the gastrointestinal lumen into the tissue and the circulatory system. Damage in intestinal integrity may cause mild to serious health issues, such as inflammation, gastrointestinal disorders, neurological diseases, and neurodegenerative disorders. Thus, maintaining a healthy intestinal barrier function is essential to sustain health. Probiotics are known for their ability to protect and restore intestinal permeability in vitro and in vivo. The multi-strain probiotics are more efficient than that of a single strain in terms of their protective efficacy. Therefore, the present study was planned and implemented to study the supplementation of probiotic mix (*Lactobacillus paracasei* HII01, *Bifidobacterium* *breve*, and *Bifidobacterium longum*) on intestinal permeability, lipid profile, obesity index and metabolic biomarkers in elderly Thai subjects. The results revealed that the supplementation of studied probiotics improved the intestinal barrier function (up to 48%), significantly increasing the high-density lipoprotein (HDL)-cholesterol. Moreover, the intervention improved obesity-related anthropometric biomarkers and short-chain fatty acid levels in human subjects. The current study strongly recommends further extended research to confirm the beneficial effect of probiotics, which may pave the way to formulate probiotic-based health supplements to adjuvant the treatment of several metabolic diseases.

## 1. Introduction

The most important protective function of the intestinal epithelium is the “barrier function,” which prevents the diffusion of allergens, toxins, and pathogens from the gastrointestinal lumen into the tissue and the circulatory system [1,2]. A specialized complex structure is present in the lateral epithelial membranes’ apical region, which is considered to be a significant module of epithelial barrier function known as tight junctions [3].

The imbalance of gastrointestinal microbiota and its function may cause interruption of the tight junctions, which provokes intestinal permeability [4]. Thus, bacterial debris, endotoxins such as lipopolysaccharides (LPS), and other microbial metabolites breach the circulatory system and reach internal organs, which can cause mild to serious health issues such as inflammation, gastrointestinal disorders, neurological diseases, and neurodegenerative disorders [5,6].

The gastrointestinal tract, especially the gut, has a complex and bidirectional communication with the central nervous system (gut–brain axis) that communicates in health and diseases [7]. The disturbance in gut microbiota might affect neurological functions and vice versa [8]. Thus, the loss of intestinal permeability might cause various neurological diseases [9,10].

Probiotics are live microorganisms, which, when administered in adequate amounts, confer health benefits [11]. Probiotics are the most recognized method to improve gut microbiota and treat dysbiosis [12,13,14]. The studies reported that the probiotics had enhanced the homeostasis of intestinal permeability [15], reduced inflammation [16], and also improved several ill-health conditions in humans [17,18]. However, the mechanism behind the beneficial effect of probiotics on health benefits is not explored. Especially in the elderly, how probiotic supplementation improves leaky gut, inflammation, and gut–brain interaction is not revealed completely and is debatable. Probiotics principally modulate gut microbiota, producing several metabolites, which confers health benefits [19].

Accordingly, the present study was planned and conducted in elderly Thai subjects to understand the impact of supplementation of a mixture of probiotics on intestinal permeability, short-chain fatty acids, markers of the gut-brain axis, and lipid profile.

## 2. Materials and Methods

### 2.1. Study Design and Participants

All the participants approved the study procedure and provided their consent before the study. The ethical committee of Mae Fah Luang University agreed to the study protocol (Code: REH-62151). The study was performed according to the Declaration of Helsinki and following the Good Clinical Practices.

The effect of a probiotics mixture on intestinal permeation and other biomarkers were studied in Thai subjects in a randomized, double-blind, placebo-controlled study model.

For the screening, subjects were asked to consume mannitol and lactulose dissolved in water. Within 6 h of mannitol and lactulose consumption, subjects were required to collect their urine [20], and their intestinal permeability was analyzed using a colorimetric commercial kit (EnzyChromTM BioAssay, San Jose, CA, USA).

Any subjects with a history of cardiovascular events, suffering from kidney diseases, gastrointestinal tract (GI) disorders, or gouty arthritis were excluded from the study. In addition, those who have undergone treatment with probiotics, antibiotic drugs (or both) or any other drugs that are used to treat GI tract-related discomforts in the previous 14 days were also excluded.

**Figure 1:**
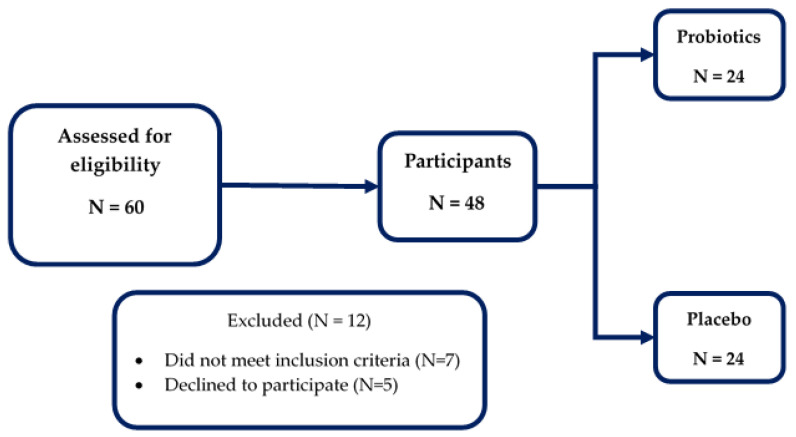
The enrollment and study flowchart.

Random Allocation Software was used to randomize the subjects, and the researchers and participants were blinded to the group assignment. The participants were randomized to receive either a probiotics supplement or placebo for 12 weeks and asked to come to the study center for follow-up. The screening and enrollment details are shown in Figure 1.

### 2.2. Treatment

The subjects in the probiotic group were provided with aluminum foil sachets containing a mixture of probiotics (2.0 × 10^10^ CFU of *Lactobacillus paracasei* HII01; 2.0 × 10^10^ CFU of *Bifidobacterium* *breve*; 1.0 × 10^10^ CFU of *Bifidobacterium longum*), which was received from Lactomason Co., Ltd., Jinju-si, South Korea, and the placebo group were provided with 10 g of corn starch in a similar package of probiotics. The instructions for the consumption of the supplement were detailed in our previous report [18].

### 2.3. Assessments

#### 2.3.1. Clinical Data

The subjects’ personal history was assessed, and their demographic characteristics were detailed. The body mass index (BMI) and weight of the subjects were measured using an electronic scale (Picooc^®^, Model S1 Pro, Beijing, China) [18].

#### 2.3.2. Laboratory Data

**Figure 2:**
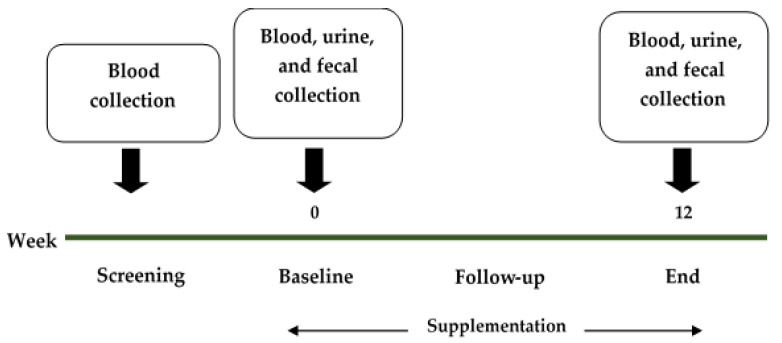
The timeline of this study, with sample collection points.

The samples (blood, urine, and feces) were collected at the screening point, baseline, follow-up, and the end of the study (Figure 2).

The biochemical parameters such as blood urea nitrogen (BUN), creatinine, aspartate aminotransferase (AST), alanine aminotransferase (ALT), total cholesterol (TC), HDL-cholesterol (HDL-C), LDL-cholesterol (LDL-C), triglycerides (TG), and fasting blood sugar (FBS) levels were measured from blood samples using the automated machine at AMS Clinical Service Center, Chiang Mai University, Chiang Mai, Thailand.

Other biomarkers in the blood, such as Immunoglobulin A (IgA) and lipopolysaccharide (LPS), were measured using ELISA commercial kit (MyBioSource^®^, San Diego, CA, USA for LPS, Elabscience^®^, Houston, TX, USA for IgA).

Urine samples were collected from subjects to determine intestinal permeability. The samples were analyzed as detailed in our previous study [18] using a colorimetric commercial kit (EnzyChrom™, BioAssay, Hayward, CA, USA). Other biomarkers in the urine, such as quinolinic acid (QA) and 5-hydroxyindoleacetic acid (5-HIAA), were determined using ELISA commercial kit (Fivephoton Biochemicals™, San Diego, CA, USA for QA, and Immusmol, Bordeaux, France for 5-HIAA).

Fecal short-chain fatty acids content was determined using high-performance liquid chromatography (HPLC) as described previously [17,21,22].

#### 2.3.3. Statistical Analyses

The data were evaluated using the paired *t*-test of means using STATA version 15.1 (StataCorp, College Station, TX, USA) for windows licensed to the Faculty of Pharmacy, Chiang Mai University.

A descriptive analysis of the collected parameters was expressed as an absolute number and percentage. The continuous variables were expressed as mean ± standard deviation (SD) or standard error of the mean (SEM), depending on their statistical distribution. The group’s data were also evaluated using Gaussian regression and Risk difference regression. The minimum level of statistical significance was set at *p* < 0.05.

## 3. Results

### 3.1. The Study Participants

A total of 60 subjects were screened, and 48 subjects were selected for randomization. According to the study design, all enrolled subjects (men: 10, women: 38) completed the trial. The primary demographic data of the study subjects are detailed in Table 1.

The subjects in placebo and probiotic groups did not significantly differe at the beginning of the study. The distribution of male and female subjects did not show any significant differences (*p* > 0.05).

### 3.2. Changes in the Study Parameters within the Group

There were no changes in study parameters after 12 weeks of the study in the placebo group except the body fat and lactulose content compared to the baseline values, whereas significant changes were observed in: body mass index (*p* = 0.010); BMR (*p* = 0.043); waist (*p* = 0.011) and hip (*p* = 0.049) circumferences; creatinine (*p* = 0.013); AST (*p* = 0.013); ALT (*p* = 0.002); HDL-C (*p* = <0.001); LDL-C (*p* = 0.001); FBS (*p* = 0.021); IgA (*p* = <0.001); LPS (*p* = 0.001); lactulose–mannitol ratio (*p* = <0.001); lactulose (*p* = 0.006); QA (*p* = <0.001); propionic acid (*p* = 0.012); and butyric acid (*p* = 0.046) content in the probiotics group after 12 weeks of intervention, when compared to baseline values. Notably, the lactulose–mannitol ratio was reduced (from 0.222 ± 0.036 to 0.047 ± 0.004) after 12 weeks of treatment. The other studied parameters were not significantly changed (Table 2).

### 3.3. Changes in the Study Parameters between the Group

Significant changes were observed in some of the study parameters in the probiotics group compared to the placebo after 12 weeks of the study. In detail, the body mass index (*p* ≤ 0.001), body fat (*p* = 0.016), muscle content (*p* = 0.022), waist (*p* = 0.001) and hip (*p* = 0.001) circumferences, creatinine (*p* = 0.001), AST (*p* = 0.024), HDL-C (*p* = 0.001), FBS (*p* = 0.001), IgA (*p* ≤ 0.001), LPS (*p* = 0.001), hsCRP (*p* = 0.029), lactulose–mannitol ratio (*p* ≤ 0.001), lactulose (*p* = 0.025), QA (*p* ≤ 0.008), and butyric acid (*p* = 0.014) content showed significant improvement in the probiotics group compared to the placebo. The results indicated that the probiotics intervention improved the study parameters in experimental subjects (Table 3).

The Gaussian regression analysis revealed that the body fat, visceral fat, muscle content, body age, arm, waist and hip circumferences, creatinine, ALT, HDL-C, FBS, IgA, LPS, lactulose–mannitol ratio, QA, QA/5-HIAA ratio, and butyric acid content were significantly altered in probiotics group after 12 weeks of treatment (Table 4). The risk difference analysis revealed that intestinal permeability was improved up to 48% in the probiotics supplemented group (Table 5).

## 4. Discussion

The study subjects completed the experimental procedures successfully and showed significant clinical improvements in intestinal permeability, lipid profile, and short-chain fatty acids.

A recent study revealed that the use of *Lactobacillus* species could improve the intestinal microbiota and reduce gut permeability [23]. According to Ohland and MacNaughton [24], probiotics improved the intestinal barrier function by increasing the production of mucus, secretory IgA and antimicrobial peptides, and increased tight junction integrity of epithelial cells and competitive adherence for pathogens.

Chen et al. [25] reported that *L. paracasei* 01 protects intestinal stability by promoting intestinal epithelial cell growth and improving intestinal integrity. Furthermore, *L. paracasei* 01 treatment inhibits the inflammatory players [tumor necrosis factor-α (TNF-α), interferon-γ (TNF-α), and C-C motif chemokine ligand-20 (CCL-20)] in vitro. Similarly, *L. paracasei* JCM 1163 also improved the intestinal barrier function via its long-chain polyphosphates accumulating property [26].

Zhang et al. [27] demonstrated that the surface-layer associated proteins (SLAP) of *L. paracasei* ssp. *paracasei* M5-L and *L. casei* Q8-L protect the bacteria-mediated epithelial barrier disruption by suppressing the occludin production and inhibiting the delocalization of zonula occludens-1. Similarly, the use of *L. paracasei* ssp. *paracasei L. casei* W8^®^ improved the intestinal barrier function and reduced the inflammation in high-fat diet-fed rats [28].

Laval et al. [29] showed that *L. rhamnosus* CNCM I-3690 enhanced intestinal integrity by increasing the level of occludin and E-cadherin.

Ahmadi et al. [19] studied the beneficial role of a human-origin probiotic (probiotics that are isolated from healthy infant gut) cocktail containing *Enterococcus* strains (*E. avium* D25-1, *E. avium* D25-2, *E. avium* D26-1, *E. raffinosus* D24-1, and *E.* INBio D24-2) and *Lactobacillus* strains (*L. paracasei* D3-5, *L. plantarum* D6-2, *L. plantarum* D13-4, *L. rhamnosus* D4-4, and *L. rhamnosus* D7-5) in aging-related leaky gut, inflammation, and metabolic dysfunctions using older C57BL/6J mice (~80 weeks mice age is equivalent to >65 years human age) as a model. The probiotic cocktail reduced physical function decline in the older mice. Furthermore, it prevented high-fat diet-induced microbiota dysbiosis by modulating the microbiota, increasing the bike salt hydrolase activity, thereby increasing the abundance of gut taurine which stimulates the tight junctions and reduces the leaky gut and inflammation [19].

Al-Sadi et al. [30] screened some probiotic species such as *Bifidobacterium bifidum, B. breve, B. longum, Escherichia coli* strain Nissle, and probiotic species or strains of *Lactobacillus acidophilus, L. brevis, L. casei, L. helveticus, L. johnsonii, L. plantarum*, and *L. rhamnosus* to identify the effective probiotic species or strain that prevents intestinal inflammation by increasing the tight junction. Among the screened probiotic bacterial species and strains, *L. acidophilus* LA1 strain showed an effective increase in Caco-2 trans-epithelial resistance and reduced paracellular permeability indicating the improvement of Caco-2 tight junction barrier function. Oral supplementation of LA1 showed TLR-2 dependent improvement of tight junction barrier and protection against intestinal inflammation, thereby preventing dextran sodium sulfate (DSS)-induced colitis in the mouse model [30].

In the present study results, the level of the lactulose-mannitol ratio and lactulose were reduced significantly in the probiotic supplemented group compared to baseline values and the placebo (Table 2 and Table 3). Moreover, the intestinal permeability of the probiotic-supplemented subjects was improved up to 48% (Table 5). The increased intestinal barrier function was observed in the serum level LPS; a significant level of reduction was observed in LPS concentration after probiotic intervention (Table 3 and Table 4). Accordingly, the studied probiotic mixture might have the ability to improve intestinal barrier function.

The reduction in the fecal concentrations of short-chain fatty acids (SCFAs) is associated with diseases and aging [31,32]. Cai et al. (2016) reported that the centenarians have a high concentration of SCFAs, associated with the high dietary fiber intake [33].

The probiotic strains of *Lactobacillus* and *Bifidobacterium* species were propionic, lactic, and butyric acid producers [34,35]. The consumption of *L. plantarum* P-8 significantly reduced the opportunistic pathogens and increased *Bifidobacterium* level. Moreover, the levels of propionate and acetate were increased [36]. Moens et al. reported that the growth of probiotic bacteria might increase the lactate concertation, which facilitates lactate-consuming microbial growth, subsequently increasing SCFAs production, particularly butyrate [37]. The further microbial analysis is required to confirm the association between changes in SCFAs levels and probiotic interventions. In the present study, probiotics intervention significantly increased propionic and butyric acid levels, whereas changes in lactic and acetic acids levels were non-significant (Table 2). Gaussian regression analysis revealed that butyric acid level increased notably (*p* = 0.008) after the probiotic intervention (Table 4).

The supplementation of *L. paracasei* HII01 (1.25 × 10^10^ CFU per day) for 12 weeks did not significantly alter IgA’s level [18]. The intervention of *L. paracasei* HII01 (5 × 10^10^ CFU per day) for 12 weeks reduced the LPS, TNF-α, IL-6, and hsCRP levels in diabetic subjects [38]. Similarly, the supplementation of synbiotic preparation (*L. paracasei* HII01, *B. longum*, *B. breve*, inulin, and fructooligosaccharide) reduced LPS, TNF-α, IL-6, and hsCRP levels. In contrast, IgA levels were increased in human subjects significantly. These results suggested that synbiotic intervention could improve obesity-associated biomarkers [17].

In the present study, BMI, body fat, muscle, and waist and hip circumferences were improved in the probiotic group compared to placebo (Table 3). The body and visceral fat, muscle, body age, and arm, waist and hip circumferences were significantly improved after the 12-week course of probiotic intervention, as per the Gaussian regression analysis (Table 4). The level of HDL-C was increased significantly in the probiotic-treated group, while a noted level of reduction was observed in the placebo (Table 3). No significant changes were observed in TC, TG, LDL-C, and hsCRP values after the study period in the probiotic-treated group (Table 4). These results indicated that the studied multi-species probiotic mix improved the lipid profile and obesity-related biomarkers in studied human subjects.

The gut microbiota and its secreted compounds may affect the tryptophan metabolism and gut inflammation, which affects the kynurenine and QA levels [39]. *L. paracasei* may influence the central 5-hydroxytryptamine (5-HT) system and brain-derived neurotrophic factor (BDNF) expression through butyrate. *B. breve* and *B. longum* affect the glutaminergic system and neural activities in the brain through humoral and neural routes, respectively [40].

The supplementation of *L. paracasei* HII01 increased the levels of short-chain fatty acids in obese, hypercholesterolemic, and diabetic human subjects [17,37]. 5-HIAA and QA/5-HIAA ratio was not significantly affected by the supplementation of *L. paracasei* HII01, *B. breve*, *B. longum*, inulin, and fructooligosaccharide. [18]. The present study results indicated that the supplementation of the studied probiotic mixture decreased the QA level (Table 3 and Table 4). Thus, the intervention might influence the tryptophan metabolism and expression of BDNF.

The probiotic intervention improved liver aminotransferases in patients with non-alcoholic fatty liver disease [41], and alcohol-induced liver disease [42]. In this study, ALT level was reduced, and AST level was not significantly changed (Table 4).

The studies on the influence of probiotics on renal function are very limited and the reported studies showed that probiotic supplementation improved renal function through increased intestinal barrier function [43,44]. In the present study also BUN values were not changed significantly, whereas creatinine levels were reduced significantly in the probiotic treated group (Table 4).

Altogether, the results of the current study provided the basic information about the influence of studied (*L. paracasei* HII01, *B. breve*, and *B. longum*) probiotic mixture on intestinal permeability, lipid profile, body fat, liver and kidney function, neurotransmitter levels, and short-chain fatty acids in elderly Thai subjects.

## 5. Conclusions

The current study has limitations, such as its limited sample size, questionnaire regarding eating habits, exercise, work activity, and overcoming the disease, lack of extended follow-up, and microbiota analysis. Nevertheless, the present study represents the effects of the intervention of a probiotic mixture composed of *Lactobacillus* and *Bifidobacterium* on kidney and liver function, lipid profile, intestinal integrity, microbial metabolites, bacterial endotoxin (LPS) level, and biomarkers of gut-brain communication pathways in elderly Thai subjects.

The results revealed that the supplementation of studied probiotics improved the intestinal barrier function, the lipid profile and obesity-related biomarkers in human subjects. Further studies are strongly recommended to confirm the beneficial effect of probiotics, which may pave the way to formulate probiotic-based health supplements to adjuvant the treatment of several metabolic diseases.

## Figures and Tables

**Table 1:** The basic characteristics of the study subjects.

Parameters	Placebo Group (*n* = 24)	Probiotics Group (*n* = 24)	*p*-Value
Age, years	58.79 ± 1.21	61.63 ± 0.84	0.061
Male, *n* (%) Female, *n* (%)	7 (29.17) 17 (70.83)	3 (12.50) 21 (87.50)	0.286
Smoking	2 (8.33)	3 (12.50)	1.000
Alcoholic	2 (8.33)	1 (4.17)	1.000
Height, cm	154.07 ± 1.57	153.40 ± 1.02	0.722
Body mass index, kg/m^2^	25.13 ± 0.66	23.95 ± 0.39	0.136
BMR, kcal	1287.96 ± 32.58	1220.27 ± 21.49	0.090
Body fat, %	29.06 ± 1.57	29.19 ± 2.43	0.965
Visceral fat, %	11.00 ± 0.90	9.38 ± 0.52	0.129
Muscle, %	66.03 ± 1.62	69.57 ± 1.20	0.086
Body age, years	59.88 ± 1.83	60.57 ± 1.03	0.747
Arm circumference, cm	28.87 ± 0.59	28.25 ± 2.75	0.827
Waist circumference, cm	86.46 ± 1.74	84.77 ± 1.23	0.433
Hip circumference, cm	97.32 ± 1.23	94.63 ± 1.11	0.112

*p*-value at 95% confidence interval. The proportions were analyzed using an exact probability test, and the continuous demographic data were analyzed using a *t*-test.

**Table 2:** Changes in the study parameters within-group at different times are expressed as mean ± SE.

Parameters	Placebo (*n* = 24)		*p*-Value	Probiotics (*n* = 24)		*p*-Value
	Baseline	12 Weeks		Baseline	12 Weeks	
Body mass index, kg/m^2^	25.13 ± 0.66	24.53 ± 1.41	0.611	23.95 ± 0.39	23.38 ± 0.34	0.010 *
BMR, kcal	1287.96 ± 32.58	1280.05 ± 28.70	0.604	1220.27 ± 21.49	1233.36 ± 19.21	0.043 *
Body fat, %	29.06 ± 1.57	32.41 ± 1.08	<0.001 *	29.19 ± 2.43	27.72 ± 1.03	0.563
Visceral fat, %	11.00 ± 0.90	11.48 ± 0.53	0.397	9.38 ± 0.52	9.14 ± 0.48	0.234
Muscle, %	66.03 ± 1.62	62.91 ± 0.61	0.079	69.57 ± 1.20	70.36 ± 1.26	0.188
Body age, years	59.88 ± 1.83	60.75 ± 1.21	0.544	60.57 ± 1.03	60.96 ± 0.94	0.464
Arm circumference, cm	28.87 ± 0.59	28.92 ± 0.61	0.883	28.25 ± 2.75	26.87 ± 0.48	0.610
Waist circumference, cm	86.46 ± 1.74	88.00 ± 1.60	0.099	84.77 ± 1.23	81.99 ± 1.26	0.011 *
Hip circumference, cm	97.32 ± 1.23	99.20 ± 1.25	0.064	94.63 ± 1.11	87.87 ± 3.49	0.049 *
BUN, mg/dL	13.07 ± 0.58	13.30 ± 0.58	0.622	13.81 ± 0.87	13.63 ± 0.99	0.811
Creatinine, mg/dL	0.81 ± 0.03	0.82 ± 0.03	0.177	0.87 ± 0.04	0.83 ± 0.03	0.013 *
AST, IU/L	23.96 ± 1.85	27.57 ± 3.57	0.352	21.60 ± 1.29	19.70 ± 1.00	0.013 *
ALT, IU/L	20.58 ± 1.68	23.42 ± 3.36	0.840	19.35 ± 1.67	16.25 ± 1.71	0.002 *
Total cholesterol, mg/dL	215.57 ± 8.48	206.35 ± 10.27	0.234	226.35 ± 9.66	217.80 ± 8.02	0.229
HDL-cholesterol, mg/dL	51.61 ± 1.76	48.22 ± 2.12	0.074	53.25 ± 2.86	56.65 ± 2.78	<0.001 *
Triglyceride, mg/dL	141.52 ± 11.81	157.17 ± 13.80	0.330	163.55 ± 21.36	147.40 ± 20.35	0.332
LDL-cholesterol, mg/dL	136.57 ± 8.02	130.10 ± 8.62	0.367	145.46 ± 7.46	126.60 ± 6.83	0.001 *
FBS, mg/dL	99.87 ± 5.85	107.09 ± 6.84	0.130	106.53 ± 8.03	98.79 ± 7.79	0.021 *
IgA, ng/mL	739.44 ± 80.41	790.20 ± 79.52	0.200	881.79 ± 50.35	1172.34 ± 50.53	<0.001 *
LPS, pg/mL	112.62 ± 16.22	94.14 ± 10.97	0.114	99.08 ± 5.10	39.82 ± 4.76	0.001 *
hsCRP, ml/L	0.0087 ± 0.0014	0.0141 ± 0.0017	0.059	0.0117 ± 0.0046	0.0060 ± 0.0020	0.201
Lactulose–Mannitol ratio	0.156 ± 0.026	0.113 ± 0.017	0.052	0.222 ± 0.036	0.047 ± 0.004	<0.001 *
Lactulose	0.1292 ± 0.0248	0.0789 ± 0.0165	0.002 *	0.0023 ± 0.0003	0.0013 ± 0.0002	0.006 *
QA, ng/mL	28.38 ± 1.83	26.49 ± 1.21	0.513	29.84 ± 0.87	19.47 ± 0.83	<0.001 *
5-HIAA, mg/L	3.17 ± 1.12	4.94 ± 1.85	0.463	8.04 ± 2.06	8.73 ± 1.38	0.551
QA/5-HIAA ratio	0.0145 ± 0.0038	0.0092 ± 0.0025	0.463	0.0056 ± 0.0009	0.0036 ± 0.0007	0.121
Lactic acid, mmol/g sample	232.96 ± 144.52	78.58 ± 22.84	0.593	48.22 ± 8.79	96.74 ± 23.06	0.066
Acetic acid, mmol/g sample	45.11 ± 0.20	37.95 ± 1.40	0.180	37.07 ± 5.25	26.94 ± 5.66	0.128
Propionic, mmol/g sample	413.81 ± 74.29	694.21 ± 216.16	0.225	411.97 ± 28.18	682.59 ± 90.31	0.012 *
Butyric acid, mmol/g sample	5.67 ± 1.02	7.47 ± 2.27	0.913	14.62 ± 5.74	63.45 ± 15.60	0.046 *

* = Significant difference in *p*-value at 95% confidence interval: AST = Aspartate aminotransferase; ALT = Alanine aminotransferase; HDL = High-Density Lipoprotein; LDL = Low-Density Lipoprotein; FBS = Fasting Blood Sugar; IgA = Immunoglobulin A; hsCRP = High Sensitivity C-Reactive Protein; LPS = Lipopolysaccharide; QA = Quinolinic acid; 5-HIAA = 5-Hydroxyindoleacetic acid.

**Table 3:** Changes in study parameters between the group at different times, expressed as mean ± SE.

Parameters	Baseline—12 Weeks		*p*-Value
	Placebo (*n* = 24)	Probiotics (*n* = 24)	
Body mass index, kg/m^2^	−0.59	−0.57	<0.001 *
BMR, kcal	−7.91	13.09	0.518
Body fat, %	3.35	−1.47	0.016 *
Visceral fat, %	0.48	−0.24	0.621
Muscle, %	−3.13	0.80	0.022 *
Body age, years	0.88	0.39	0.324
Arm circumference, cm	0.05	−1.38	0.137
Waist circumference, cm	1.55	−2.78	0.001 *
Hip circumference, cm	1.88	−6.77	0.001 *
BUN, mg/dL	0.23	−0.18	0.752
Creatinine, mg/dL	0.02	−0.04	0.001 *
AST, IU/L	3.61	−1.90	0.024 *
ALT, IU/L	2.84	−3.10	0.055
Total cholesterol, mg/dL	−9.22	−8.55	0.670
HDL-cholesterol, mg/dL	−3.39	3.40	0.001 *
Triglyceride, mg/dL	15.65	−16.15	0.154
LDL-cholesterol, mg/dL	−6.46	−18.86	0.173
FBS, mg/dL	7.22	−7.74	0.001 *
IgA, ng/mL	50.76	290.55	<0.001 *
LPS, pg/mL	−18.48	−59.26	0.001 *
hsCRP, ml/L	0.005	−0.006	0.029 *
Lactulose–Mannitol ratio	−0.04	−0.18	0.001 *
Lactulose	−0.0502	−0.0010	0.025 *
QA, ng/mL	−1.89	−10.36	0.008 *
5-HIAA, mg/L	1.77	0.69	0.837
QA/5-HIAA ratio	−0.005	−0.002	0.461
Lactic acid, mmol/g sample	−154.38	48.53	0.079
Acetic acid, mmol/g sample	−7.16	−10.12	0.558
Propionic, mmol/g sample	280.40	270.62	0.965
Butyric acid, mmol/g sample	1.79	48.83	0.014 *

* = Significantly difference in *p*-value at 95% confidence interval, AST = Aspartate aminotransferase; ALT = Alanine aminotransferase; HDL = High-Density Lipoprotein; LDL = Low-Density Lipoprotein; FBS = Fasting Blood Sugar; IgA = Immunoglobulin A; hsCRP = High Sensitivity C-Reactive Protein; LPS = Lipopolysaccharide; QA = Quinolinic acid; 5-HIAA = 5-Hydroxyindoleacetic acid.

**Table 4:** Gaussian regression analysis summary at week 12 of treatment for probiotics group.

Parameter	Coefficient	95% CI	*p*-Value
Body mass index, kg/m^2^	−0.86	−4.35 to 2.62	0.612
BMR, kcal	−9.38	−35.21 to 16.45	0.458
Body fat, %	−3.65	−4.76 to −2.54	<0.001 *
Visceral fat, %	−0.84	−1.41 to −0.28	0.006 *
Muscle, %	4.23	1.83 to 6.62	0.001 *
Body age, years	−2.31	−4.07 to −0.54	0.012 *
Arm circumference, cm	−2.35	−3.99 to −0.70	0.007 *
Waist circumference, cm	−3.74	−7.07 to −0.42	0.029 *
Hip circumference, cm	−5.47	−9.96 to −0.97	0.019 *
BUN, mg/dL	−0.75	−2.89 to 1.38	0.477
Creatinine, mg/dL	−0.04	−0.076 to −0.003	0.033 *
AST, IU/L	−7.96	−16.60 to 0.67	0.069
ALT, IU/L	−8.27	−15.56 to −0.99	0.028 *
Total cholesterol, mg/dL	6.65	−15.51 to 28.80	0.546
HDL-cholesterol, mg/dL	7.62	2.80 to 12.44	0.003 *
Triglyceride, mg/dL	−18.13	−52.47 to 16.22	0.290
LDL-cholesterol, mg/dL	−1.42	−19.92 to 17.09	0.877
FBS, mg/dL	−13.63	−25.72 to −1.54	0.028 *
IgA, ng/mL	230.18	76.39 to 383.98	0.005 *
LPS, pg/mL	−58.03	−82.59 to −33.46	<0.001 *
hsCRP, ml/L	−0.008	−0.020 to 0.004	0.147
Lactulose–Mannitol ratio	−0.08	−0.12 to −0.04	0.001 *
Lactulose	−0.004	−0.030 to 0.021	0.733
QA, ng/mL	−6.97	−10.17 to −3.77	0.001 *
5-HIAA, mg/L	3.43	−3.81 to 10.68	0.307
QA/5-HIAA ratio	−0.01	−0.02 to −0.01	0.002 *
Lactic acid, mmol/g sample	60.65	−279.10 to 400.41	0.610
Acetic acid, mmol/g sample	−9.85	−89.82 to 70.12	0.649
Propionic, mmol/g sample	−19.43	−466.59 to 427.72	0.925
Butyric acid, mmol/g sample	47.79	14.54 to 81.04	0.008 *

* = Significant difference in *p*-value at 95% confidence interval. Comparison with placebo group at week 12: AST = Aspartate aminotransferase; ALT = Alanine aminotransferase; HDL = High-Density Lipoprotein; LDL = Low-Density Lipoprotein; FBS = Fasting Blood Sugar; IgA = Immunoglobulin A; hsCRP = High Sensitivity C-Reactive Protein; LPS = Lipopolysaccharide; QA = Quinolinic acid; 5-HIAA= 5-Hydroxyindoleacetic acid.

**Table 5:** Risk difference analysis of probiotics treatment.

Parameter	Risk Difference	95% CI	*p*-Value
Leaky gut	−0.48	−0.79 to −0.18	0.002 *

* = Significant difference in *p*-value at 95% confidence interval. Comparison with placebo group at week 12.

## Data Availability

The data presented in the manuscript is available on request from the corresponding author.
